# Regional forest stock volume mapping using GEDI-based interpolation, multi-source remote sensing, and a multi-level stacking ensemble model in complex terrain

**DOI:** 10.3389/fpls.2026.1827732

**Published:** 2026-04-30

**Authors:** Zeyu Li, Qingtai Shu, Lianjin Fu, Rong Wei, Yiran Zhang, Xiao Zhang, Siqi Meng

**Affiliations:** 1 College of Soil and Water Conservation, Southwest Forestry University, Kunming, China; 2 College of Forestry, Southwest Forestry University, Kunming, China

**Keywords:** complex terrain, FSV, GEDI, multi-level ensemble stacking model, spatial interpolation

## Abstract

**Introduction:**

Forest Stock Volume (FSV) is a key indicator of forest ecosystem functions, including productivity and carbon storage. Although spaceborne LiDAR such as GEDI provides high-resolution information on forest vertical structure, its discrete sampling pattern poses challenges for generating continuous spatial representations.

**Methods:**

This study developed an integrated framework for FSV estimation in *Pinus kesiya* var. *langbianensis* forests in Jingdong County, Yunnan, China, by combining GEDI LiDAR metrics, Landsat-8 imagery, topographic variables, and field measurements from 143 sample plots. Six spatial interpolation methods were compared to transform discrete GEDI observations into continuous predictor layers. A multi-level stacking ensemble model (MLSEM) integrating six base machine-learning algorithms was then constructed for FSV estimation.

**Results:**

Among the tested interpolation methods, Sequential Gaussian Conditional Simulation (SGCS) performed best, and the SGCS-derived variables generally achieved R^2^ values greater than 0.50. The final stacked model outperformed all base learners and single-level meta-learners, achieving an R^2^ of 0.93 and an RMSE of 9.50 m^3^/ha. The total standing stock in 2019 was estimated at approximately 8.66 × 10^7^ m^3^, with a mean volume of 43.89 m^3^/ha.

**Discussion:**

These results demonstrate the value of combining interpolated GEDI structural information with multi-source remote-sensing predictors for regional FSV mapping in complex mountainous terrain. The resulting spatially explicit FSV map can provide useful support for forest inventory updating, productivity monitoring, and management prioritization.

## 1 Introduction

Forest Stock Volume (FSV) is a key parameter for evaluating forest carbon sequestration capacity (Liu et al., 2022b). It is also an important indicator of forest resource status and management level (Mendoza and Prabhu, 2003; Sasaki et al., 2021; Li et al., 2022). Its estimation generally relies on both remote sensing and ground-based surveys (Sarker and Nichol, 2011). However, traditional field surveys are often labor-intensive, costly, and difficult to implement in remote or inaccessible regions (Kumar and Mutanga, 2017). Consequently, remote sensing has become an essential tool for regional-scale FSV mapping (Liu et al., 2023). Nevertheless, reliable wall-to-wall FSV estimation remains challenging because forest structure varies markedly across space (Hui et al., 2019), and many remote-sensing signals exhibit nonlinear relationships with forest structural parameters (Frolking et al., 2009).

Optical satellite imagery has long been used for forest attribute retrieval because of its broad spatial coverage and long-term data archives (Chrysafis et al., 2017; Mulverhill et al., 2025; Zhao and Yu, 2025). A common limitation is spectral saturation in high-volume stands, which reduces sensitivity and often leads to biased estimates (Wulder et al., 2010; Zhao et al., 2016; Ou et al., 2019). In particular, this effect typically results in the underestimation of high FSV values and the overestimation of low FSV values (Hu et al., 2020; Ma et al., 2023; Li et al., 2024b). To mitigate these limitations, techniques such as spatial regression models (Yu et al., 2023) and multi-source data fusion (Wang et al., 2024a; Yang et al., 2025b) have been employed. Nevertheless, optical observations remain primarily two-dimensional and cannot directly characterize vertical canopy structure, which is essential for estimating volume-related forest attributes (Lausch et al., 2016).

LiDAR provides direct measurements of forest vertical structure and has demonstrated strong performance in estimating forest structural attributes from plot to regional scales (Hilbert and Schmullius, 2012; Pourrahmati et al., 2015; Song et al., 2022; Wang et al., 2024b; Yang et al., 2025a; Zhang et al., 2025). Airborne LiDAR achieves high accuracy but is expensive and difficult to acquire repeatedly over large regions (Meng et al., 2010; Xiang et al., 2019). In contrast, spaceborne LiDAR offers broader coverage and greater repeatability (Wulder et al., 2012). In particular, the Global Ecosystem Dynamics Investigation (GEDI) mission provides a dedicated spaceborne LiDAR dataset designed to measure forest canopy height, vertical structure, and ground elevation (Xu et al., 2024). Accordingly, fusing GEDI-derived structural metrics with optical remote-sensing features has become an important strategy for improving the estimation accuracy of forest structural parameters, and recent studies have demonstrated the effectiveness of this approach for large-scale forest resource mapping. For instance, Potapov et al. (2021) integrated GEDI data with Landsat imagery to map forest canopy height at a global scale. Tamiminia et al. (2024) fused GEDI footprints with Sentinel-2 imagery to produce a 10 m wall-to-wall canopy height model (CHM) for New York State in 2019 and subsequently combined the CHM with Sentinel-1/2 data to map aboveground biomass (AGB) at the same spatial resolution. Luo et al. (2024) combined GEDI structural metrics with Landsat-derived predictors to generate a continuous wall-to-wall biomass map for the Shangri-La region of Yunnan Province, achieving an R^2^ of 0.82 and an RMSE of 35.51 t/ha.

Stacked generalization is an ensemble learning strategy that can improve predictive stability and accuracy by combining the predictions of diverse base learners through a meta-learner (Du et al., 2021; Hu et al., 2021; Li et al., 2021; Jiang et al., 2025a). In multi-source remote sensing, simple feature concatenation may fail to fully exploit the nonlinear complementarities among heterogeneous predictor groups (Silva et al., 2021). In recent years, stacking approaches have been increasingly applied to the estimation of forest structural attributes and have often shown greater robustness than individual models under complex stand conditions or multi-source data integration scenarios (Jin et al., 2025; Liu et al., 2025). Xu et al(2025) reported that ensemble learning methods exhibited better fitting ability and stability under complex forest stand conditions. Jiang et al.(2021) used ICESat-2 LiDAR data to construct a stacking model for temperate forest canopy height estimation, demonstrating that the integration of different base learners can improve predictive performance. However, most existing studies have focused on direct predictor fusion or single-level stacking architectures, whereas relatively few have explored the combined use of GEDI-derived structural information, interpolation strategy comparison, and a multi-level stacking framework for forest stock volume inversion in complex mountainous regions. Based on this background, we hypothesize that a multi-level stacking architecture, by hierarchically integrating intermediate predictions from multiple learning stages, can further enhance the generalization ability and robustness of regional FSV estimation in complex terrain.

Despite these advances, two major bottlenecks remain in regional FSV estimation based on GEDI data fusion. First, GEDI observations are spatially discrete footprints, and wall-to-wall mapping therefore requires footprint-derived metrics to be transformed into continuous surfaces through spatial modeling or interpolation (Xu et al., 2023; Xia et al., 2024). Second, the choice of interpolation strategy, together with its associated uncertainty, can substantially affect the derived GEDI predictor layers and, consequently, downstream machine-learning performance. However, many existing studies rely on a single interpolation method without systematically evaluating how interpolation choices influence final prediction accuracy and spatial patterns. In addition, most existing studies rely on individual machine-learning models or single-level integration strategies, while relatively few have explored whether hierarchical ensemble frameworks can more effectively integrate GEDI-derived structural predictors with optical and topographic variables for regional FSV estimation in complex mountainous terrain.

To address these gaps, this study focuses on *Pinus kesiya* var. *langbianensis* forests in Jingdong County, Yunnan Province, China, and proposes an integrated framework for regional FSV mapping. This study was guided by the following research questions:

1. Which interpolation strategy is most effective for transforming discrete GEDI footprints into continuous predictor layers in mountainous forests?
2. Can a multi-level stacking ensemble model outperform individual machine-learning models and single-level stacking models in regional FSV estimation?
3. Does the integration of interpolated GEDI structural metrics with optical and topographic predictors improve the robustness of FSV mapping in complex terrain?

Accordingly, we hypothesized that:

1. Interpolation methods that better preserve spatial heterogeneity would generate more informative GEDI-derived predictor layers;
2. The MLSEM would outperform individual base learners and single-level stacking models by more effectively exploiting nonlinear complementarities among heterogeneous predictors;
3. Integrating GEDI-derived structural information with optical and topographic variables would improve the robustness of regional FSV estimation in mountainous terrain.

The objective of this study was to evaluate interpolation strategy effects and develop a hierarchical ensemble framework for robust regional FSV estimation in mountainous terrain.

## 2 Materials and methods

### 2.1 Study area

**Figure 1:**
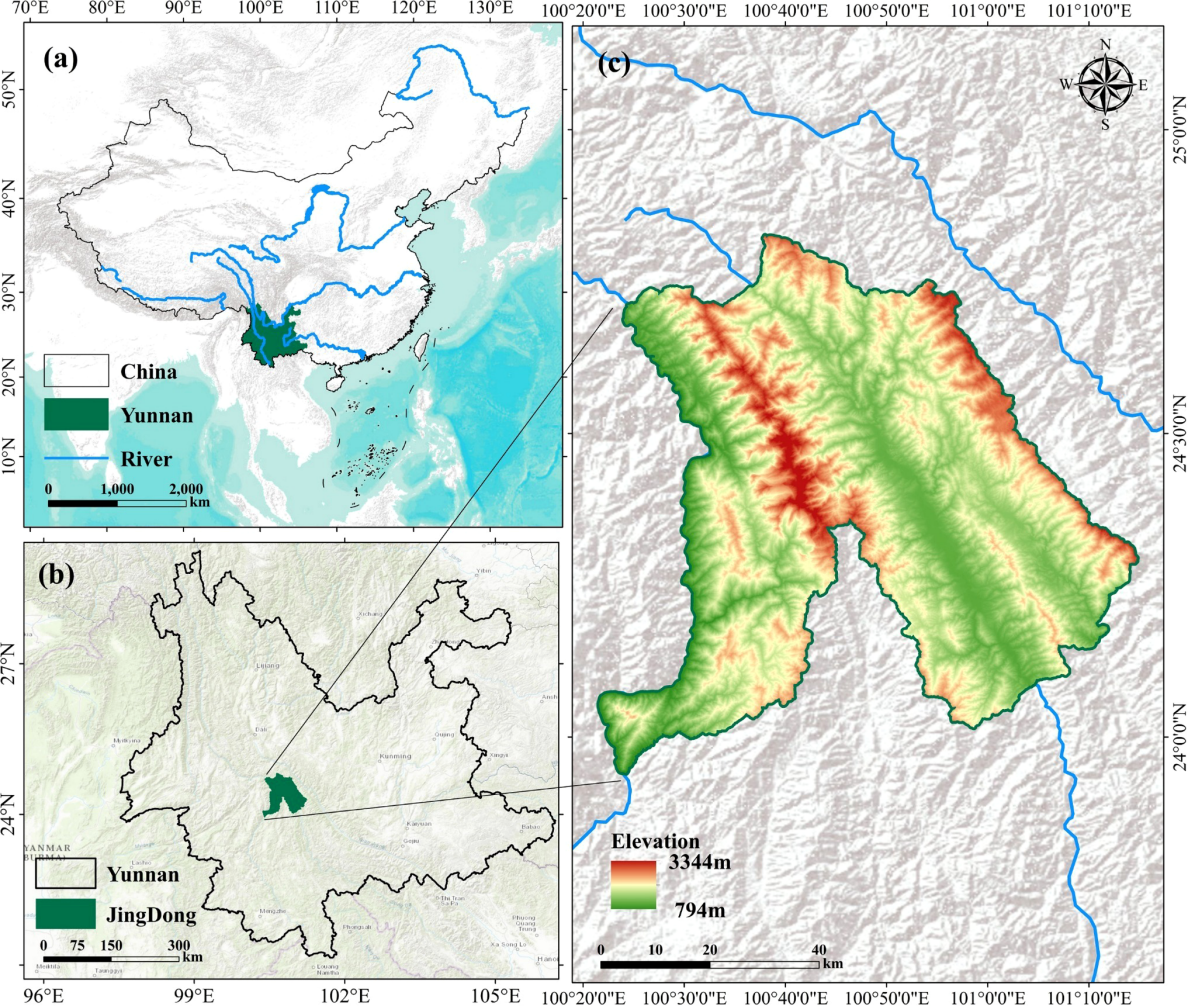
Geographic location of the study area. **(A)** Shows the standard map of China (Map Examination No.: GS (2024) 0650); **(B)** Shows the administrative boundary of Jingdong County (Map Examination No.: Yun S (2025) 104); and **(C)** shows the DEM and the distribution of field sample plots in Jingdong County. The DEM data were derived from the Shuttle Radar Topography Mission (SRTM).

As shown in **Figure 1**, Jingdong Yi Autonomous County is located at the southern terminus of the Hengduan Mountains and is dominated by the north-south-oriented Wuliang and Ailao mountain systems. This topographic setting has resulted in a deeply dissected mid-mountain landscape characterized by high ridges, steep slopes, and deep valleys. Elevation ranges from 794 to 3,344 m, resulting in pronounced topographic heterogeneity. Forest composition in the study area is also highly diverse, including *Pinus kesiya* var. *langbianensis*, oaks, *Alnus cremastogyne Burkill*, *Eucalyptus urophylla S.T.Blake*, *Cunninghamia lanceolata (Lamb.) Hook.*, *Pinus armandii Franch.*, *Betula L.*, *Schima superba Gardner & Champ.*, *Pinus yunnanensis Franch.*, *Tsuga chinensis (Franch.) E. Pritz.*, and other broadleaved species. Together, the complex topography and diverse forest composition make this region well suited for evaluating multi-source remote-sensing-based FSV estimation approaches under challenging environmental conditions.

### 2.2 Data collection

#### 2.2.1 Field data collection and forest stock volume estimation

**Figure 2:**
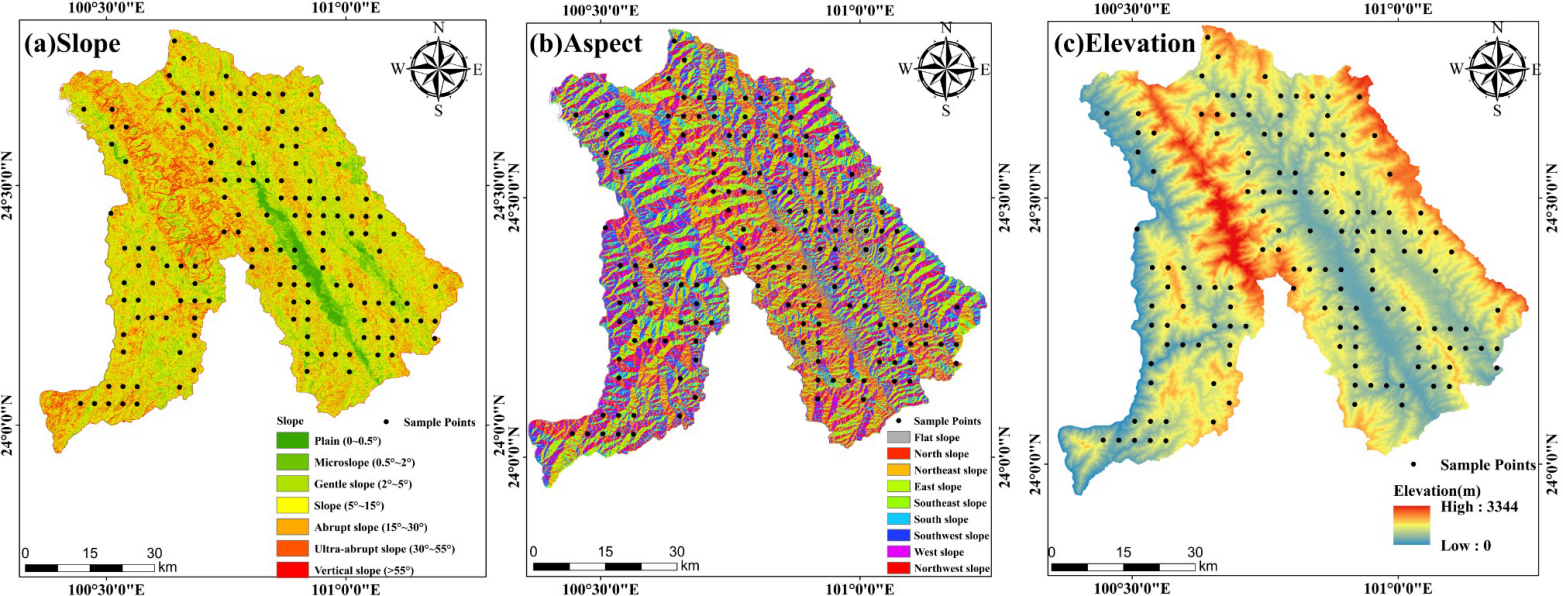
Slope **(A)**, Aspect **(B)** and Elevation **(C)** of the study area.

Field data were sourced from the Second Forest Resources Survey of Jingdong County (2016) and 143 one-hectare *Pinus kesiya* var. *langbianensis*, one of the most widely distributed and representative tree species in the study area. These plots were distributed across representative elevational gradients (794–3344 m), slope classes (plain, micro slope, gentle slope, slope, abrupt slope, ultra-abrupt slope, and vertical slope), and aspects. For each plot, geographic coordinates, land-cover type, dominant species, mean diameter at breast height (DBH), and mean tree height were recorded, and stand volume was derived from a species-specific two-entry volume table. Prior to analysis, all plots were verified in the field to confirm site conditions and data usability. The final dataset used in this study included all 143 plots (**Figure 2**), providing an adequate sample size for the subsequent statistical analyses.

To reduce temporal mismatch between the plot observations and the remote-sensing predictors, we implemented a two-step preprocessing workflow. First, plot-level stand volume measured in 2016 was projected to 2019 using a localized growth equation for *Pinus kesiya* var. *langbianensis* natural secondary forest in Yunnan (Xu, 2001), and the Stand Density Index (SDI) was calculated using Equation (1). The stand-volume growth model used for temporal projection is shown in Equation (2). According to the original study, the stand-volume growth model reported a low average relative error (E = 0.064%), indicating a relatively small model error within its applicable range. Because no independent field survey was available in 2019, however, the projected values could not be directly validated against contemporaneous field measurements and should therefore be regarded as a pragmatic adjustment for temporal harmonization rather than independently validated ground truth. Uncertainty introduced during this projection process may still have propagated into model calibration and the final FSV estimates.

Second, to improve comparability between the field-based response variable and the 30 m mapping unit used for regional prediction, stand volume was converted from a per-hectare basis to an areal basis corresponding to the 30 m grid. Specifically, because one 30 m × 30 m pixel represents 900 m^2^ whereas one hectare equals 10,000 m^2^, plot-based FSV was converted using an areal scaling factor of 900/10,000. This conversion was intended as unit harmonization within the mapping framework rather than as a strict support-domain transformation. Therefore, although it improves comparability with the map unit, it does not imply that support mismatch among the 1-ha field plots, GEDI footprints, and 30 m map pixels was fully eliminated. A statistical summary of the field plot data used in this study is provided in **Table 1**.

**Table 1:** Statistical summary of FSV in *Pinus kesiya* var. *langbianensis* sample plots.

Statistical parameters	Number	Minimum/(m³/ha)	Maximum value/(m³/ha)	Mean value/(m³/ha)	Standard deviation/(m³/ha)	Coefficient of variation
Value	143	2.56	273.80	77.7885	64.5086	0.8306

(1) $$\;SDI=407181.974\times {\left(D_{0}/D\right)}^{-2.209}$$

(2) $$V^{\prime }=a_{1}•SI^{a_{2}}/[1-a_{3}•SDI^{a_{4}}•EXP(-a_{5}•t)]$$

In Formula 1, *SDI* denotes the stand density index; $D_{0}$ represents the stand standard diameter; $D_{0}$*=12* cm, denotes the average diameter of the sample plot. In Formula 2, *SI* stands for the site index, with its average value taken as *SI=18.39*; the values of the other parameters are as follows: $a_{1}=17.6139$, $\;a_{2}=0.06798776$, $a_{3}=-0.39435932$, $a_{4}=-1.5839$, $a_{5}=0.0906281$.

#### 2.2.2 GEDI data

The GEDI instrument acquires high-resolution laser ranging observations of the Earth’s three-dimensional structure, thereby generating datasets for different applications. GEDI data products are categorized into four progressive processing levels (L1-L4) (Zhu, 2021). GEDI L2B version 2 data acquired between April and December 2019 were used, providing geolocated elevation and vertical structure metrics, including relative height (RH) percentiles. Each footprint has a diameter of approximately 25 m, with an along-track spacing of about 60 m between consecutive footprints and about 600 m between adjacent tracks (Dubayah et al., 2020). The extracted metrics are listed in **Table 2**. No additional empirical correction for horizontal displacement was applied beyond the standard geolocation information provided in the GEDI L2B product. Given the improved geolocation performance of Version 2, this product is considered suitable for many scientific applications that can tolerate moderate geolocation error (Tang et al., 2023).

**Table 2:** Description of GEDI L2B parameters used for modeling and analysis.

Name	Descriptive
Cover (%)	Canopy cover
Fhd_normal	Leaf height diversity index
Elevation (m)	Digital elevation model provided by GEDI
Pai	Plant area index
Pgap_theta	vegetation gap
Rg (m)	Integration of the ground component of a waveform
Rh98 (m)	Relative Height 98th percentile
Rv	Integration of the vegetation component of the waveform
Sensitivity (%)	Maximum canopy cover penetrable by waveforms
Landsat_treecover (%)	Tree cover from Landsat data
Leaf_off_doy (DOY)	Data without vegetation
Leaf_on_doy (DOY)	Data with vegetation
Modis_nonvegetated (%)	Percent unvegetated standard deviation of MODIS data
Modis_treecover (%)	Tree cover from MODIS data
quality_flag	Signal Quality Marker

To ensure data quality, a series of filters consistent with established methodologies (Han et al., 2022), were applied to the raw GEDI waveforms. The filtering criteria were as follows: (1) lat_lowestmode and lon_lowestmode were used to determine footprint locations; (2) only footprints with sensitivity values greater than 0.9 were retained; and (3) quality_flag = 1 was required to ensure that the waveform satisfied the recommended quality-control standards. After quality screening, a total of 67,072 high-quality GEDI footprints were retained, including 50,096 classified as forest and 16,976 classified as non-forest. Subsequent analyses focused on the forest footprints (n = 50,096).

#### 2.2.3 Landsat-8 image data

The Landsat 8 satellite carries a dual-sensor payload, consisting of the Operational Land Imager (OLI) and the Thermal Infrared Sensor (TIRS) (Irons et al., 2012). Landsat 8 Collection 2 Level-2 surface reflectance imagery (30-m spatial resolution) for the study area was accessed and processed in Google Earth Engine (GEE). To reduce contamination from atmospheric and surface artifacts, we applied a quality-assurance (QA) mask based on the QA_PIXEL band to remove clouds (bit 5), cloud shadows (bit 3), and snow (bit 4), thereby retaining only clear observations. Surface reflectance values were further converted to physical reflectance using the official scaling factors (scale factor = 0.0000275; additive offset = -0.2). All available images acquired between January and December 2019 were then aggregated into a single annual composite using the median reducer, which effectively suppresses residual outliers and improves spatial completeness in cloudy mountainous regions.

From the 2019 composite, 11 vegetation indices and 8 texture features were derived. Texture features were computed from the gray-level co-occurrence matrix (GLCM) using the near-infrared (NIR) band with a 5×5 window, a one-pixel displacement, and 64 grayscale quantization levels (Cai et al., 2020). The NIR band was selected because it is sensitive to vegetation canopy structure and density, thereby enabling more effective characterization of spatial heterogeneity while maintaining relatively stable and noise-robust estimates at 30 m. A 5×5 window was adopted as a practical compromise for capturing local canopy heterogeneity while limiting excessive smoothing and noise sensitivity at this spatial resolution, given that the optimal GLCM window size is not universal but depends on sensor resolution, target variables, and study conditions. Explicit topographic correction was not applied, and the potential effects of terrain-induced illumination variation in this rugged landscape are discussed as a limitation. All derived vegetation indices and texture features are listed in **Table 3**.

**Table 3:** Description of Landsat-8 parameters used for modeling and analysis.

Name	Descriptive
NGBDI (Verrelst et al., 2008)	Normalized Blue-Green Difference Index
ARVI (Kaufman and Tanre, 1992)	Atmospheric Resistance Vegetation Index
NDVI (Huang et al., 2021)	Normalized Difference Vegetation Index
DVI (Donovan et al., 2022)	Difference Vegetation Index
EVI (Ou et al., 2019)	Enhanced Vegetation Index
TVI (Qian et al., 2022)	Triangle Vegetation Index
SAVI (Huete, 1988)	Soil-Adjusted Vegetation Index
RVI (Zeng et al., 2022)	Ratio Vegetation Index
VDVI (Wang et al., 2015)	Visible - band Difference Vegetation Index
NPCI (Clay et al., 2006)	Chlorophyll Normalized Vegetation Index
RDVI (Roujean and Breon, 1995)	Renormalized Difference Vegetation Index

Although Sentinel-2 can outperform Landsat-8 in some forest applications because of its finer spatial resolution and red-edge bands (Chrysafis et al., 2017), Landsat-8 surface reflectance was used in this study to ensure temporal consistency with the target year (2019), maintain a uniform 30 m analysis grid for integrating terrain and texture predictors, and achieve spatially continuous coverage over the mountainous study area. An annual median composite was adopted rather than a single-season image because frequent cloud cover and topographic shadow substantially reduce the spatial completeness of season-specific imagery in this region. While this strategy may smooth seasonal phenological differences, it improves wall-to-wall coverage and was considered acceptable for stand-level FSV estimation.

#### 2.2.4 Digital elevation model data

Topographic variables, including slope, aspect, and elevation, were derived from the 30 m Shuttle Radar Topography Mission (SRTM) Digital Elevation Model (DEM) using the Spatial Analyst module in ArcMap 10.8, as illustrated in **Figure 2**.

### 2.3 Research methods

**Figure 3:**
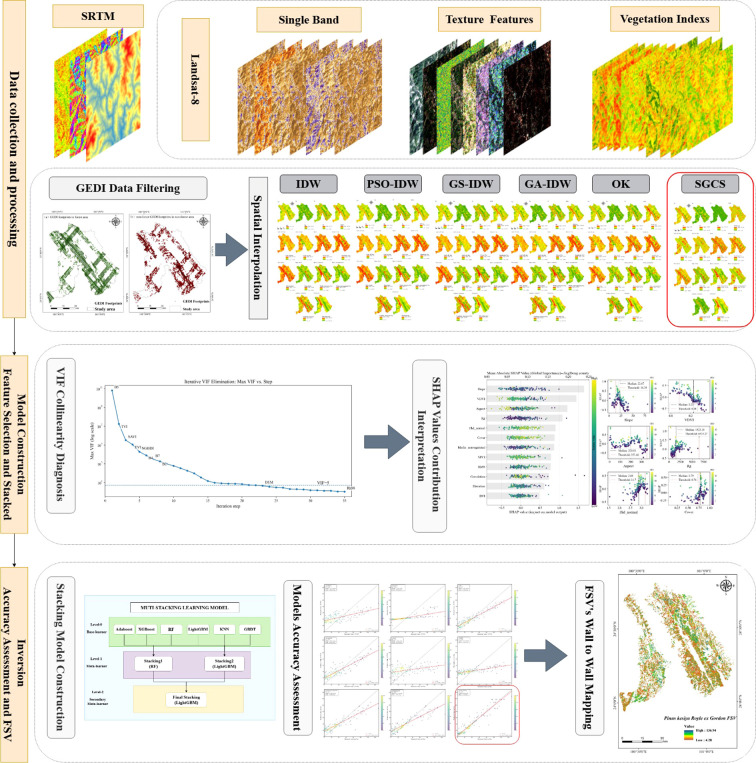
Overall technical workflow of the study.

The overall technical workflow of this study is illustrated in **Figure 3** and can be summarized as follows:

1. Multi-source data were collected and processed, including Landsat-8 imagery, SRTM-derived topographic variables, GEDI LiDAR data, and field plot measurements. From Landsat-8, spectral bands, vegetation indices, and GLCM-based texture features were derived, while slope, aspect, and elevation were extracted from the DEM.
2. GEDI footprints were quality filtered, and multiple spatial interpolation methods, including inverse distance weighting (IDW), particle swarm optimization-based IDW (PSO-IDW), grid search-based IDW (GS-IDW), genetic algorithm-based IDW (GA-IDW), ordinary kriging (OK), and sequential Gaussian conditional simulation (SGCS), were compared to transform discrete GEDI structural metrics into continuous raster predictor layers.
3. Predictor variables were screened using a two-stage feature selection procedure, in which multicollinearity was first reduced using VIF and the remaining variables were then ranked using SHAP values. The selected variables were used to construct MLSEM.
4. Model performance was evaluated by comparing the MLSEM with the candidate base learners and meta-learners, and the optimized model was finally applied to generate a wall-to-wall FSV map for the study area.

#### 2.3.1 Variational functions

The variogram is a foundational tool in spatial statistics for quantifying the spatial autocorrelation inherent in geographic data (Schabenberger and Gotway, 2017). In forest ecosystems, this autocorrelation arises from key ecological processes, including resource competition, natural disturbances, and regeneration dynamics, which impose structured spatial dependence on FSV (Marta et al., 2019; Zhang et al., 2022). The variogram characterizes the spatial variation of regional-scale variables by fitting theoretical models, such as the spherical, exponential, and Gaussian models (Wang et al., 2022), which are defined by three core parameters: the nugget (*C_0_*), sill (*C_0_+C*), and range (Xu et al., 2016). The range is the distance at which the variogram stabilizes and reaches the sill, indicating the maximum extent of spatial correlation. The nugget (*C_0_*) represents the non-zero semi-variance at zero distance and reflects sampling error and small-scale variability. The degree of spatial dependence is commonly assessed using the nugget-to-sill ratio, *C_0_/C_0_+C*: values < 25% indicate strong spatial dependence, values between 25% and 75% indicate moderate spatial dependence, and values > 75% indicate weak spatial dependence (Yu et al., 2024).

Empirical variograms for the GEDI LiDAR metrics were fitted in GS + 9.0 using spherical, exponential, and Gaussian models. Model adequacy was evaluated using R^2^ and RSS, and the best-fitting model was retained. The selected variogram type and its associated parameters (nugget, sill, and range) were then used in the subsequent Ordinary Kriging (OK) interpolation and Sequential Gaussian Conditional Simulation (SGCS).

#### 2.3.2 Spatial interpolation of LiDAR metrics

To transform discrete GEDI footprints into continuous raster predictor layers, six spatial interpolation methods were evaluated in this study: inverse distance weighting (IDW), particle swarm optimization-based IDW (PSO-IDW), grid search-based IDW (GS-IDW), genetic algorithm-based IDW (GA-IDW), ordinary kriging (OK), and sequential Gaussian conditional simulation (SGCS).

OK estimates values at unsampled locations as a weighted average of neighboring observations, with weights derived from the variogram model to ensure unbiased estimates with minimum variance (Oliver and Webster, 1990). In this study, OK was implemented in ArcMap 10.8 using the variogram model type and associated parameters previously fitted in GS + 9.0. The OK predictor at location $S_{0}$ is given by Equation (3):

(3) $$Z(S_{0})=\sum_{i=1}^{n}\lambda_{i}Z(S_{i})$$

In the formula, $Z(S_{i})$ is the measured value of the *i-th* position, $\lambda_{i}$ is the unknown weight of the measured value of the *i-th* position, $S_{0}$ is the predicted position.

SGCS was employed to generate multiple realizations of the spatial field, thereby preserving the spatial variability characterized by the variogram model (Luo et al., 2023). Unlike OK, which produces a single smoothed estimate, SGCS generates simulated values by sampling from the conditional distribution at each unsampled location. In practice, the variogram defines the spatial dependence structure used to derive the kriging-based conditional mean and variance from neighboring observations, after which a stochastic residual term is introduced to generate simulated values that honor both the observed data and the target variogram. For a location $X_{0}$, a common formulation is:


(4) $$\text{Y}({\text{X}}_{0})=\text{μ}+\sum_{\text{i}=1}^{\text{n}}{\text{λ}}_{\text{i}}\cdot \text{Y}({\text{X}}_{\text{i}})+\in ({\text{X}}_{0})$$

where μ is the mean value of the predicted position, $Y(X_{i})$ are neighboring observed values, $\lambda_{i}$ are the unknown weight of the measured value of the *i*-th position, n is the number of conditioning neighbors, and $\in (X_{0})$ is a stochastic residual drawn such that the realizations reproduce the spatial dependence characterized by the variogram.

IDW was applied, with sample weights assigned inversely proportional to distance (Wagner et al., 2012). The distance exponent ( β) was optimized for each variable using PSO, GA, and GS, with the objective of minimizing the root mean square error (RMSE) obtained from five-fold cross-validation. Specifically, PSO was run with a swarm size of 15, 50 iterations, an inertia weight of ω=0.8, and acceleration coefficients of $c_{1}=c_{2}=1.5$The value of β was initialized within the range [0.1,5.0] and constrained to [0.1,10.0].The GA used a population size of 15, 50 generations, a crossover probability of $p_{c}=0.8$, a mutation probability of $p_{m}=0.1$, and the same bounds for β. GS evaluated β from 0.5 to 5.0 with a step size of 0.1, and the value yielding the lowest RMSE was selected.

The 50,096 GEDI footprints were divided into a training set (80%) for spatial interpolation and a hold-out test set (20%) for independent evaluation of interpolation performance. The interpolated surfaces of each LiDAR structural metric were then resampled in ArcGIS to continuous raster layers at 30 m spatial resolution to match the Landsat-8 and DEM predictors. Specifically, the output cell size was set to 30 m, the output extent was kept consistent with the input raster, and the nearest-neighbor method was used to harmonize the grid while avoiding the creation of new intermediate values during resampling. Given that the GEDI footprint diameter is approximately 25 m, this preprocessing step was considered reasonable for multi-source predictor fusion, although it does not fully remove support mismatch or geolocation-related uncertainty. Interpolation performance was evaluated using the coefficient of determination (R^2^), as defined in Equation (5).

(5) $$R^{2}=\frac{|\sum_{i=1}^{N}\hat{Z}(x_{i})-\bar{Z}(x_{i}){|}^{2}}{|\sum_{i=1}^{n}Z(x_{i})-\bar{Z}(x_{i}){|}^{2}}$$

Where $\hat{Z}(x_{i})$ is the predicted value at location $x_{i}$, $Z(x_{i})\;$is the observed value at location $x_{i}$, $\bar{Z}(x_{i})$ is the mean of the observed values $x_{i}$, and *n* is the total number of samples.

#### 2.3.3 Feature selection

A two-stage feature-selection procedure was employed. First, multicollinearity was reduced by excluding features with a variance inflation factor (VIF) > 5, because values at or above this level are widely regarded as indicating potentially problematic multicollinearity in regression analysis, which may inflate variance and reduce the interpretability of the retained predictors (Akinwande et al., 2015). Second, SHapley Additive exPlanations (SHAP) values were computed using an RF model to rank the global importance of the remaining predictors. RF was selected because it can capture nonlinear relationships and provides stable SHAP-based interpretations for tree-based models under limited sample sizes. Predictors were ranked according to their mean absolute SHAP values, and the top six were retained to form a parsimonious and interpretable feature set that reduces the risk of overfitting while maintaining predictive performance (Li et al., 2024a).

#### 2.3.4 FSV estimation using stacked ensemble modeling

Because the relationships among topographic and vegetation predictors in the study area are highly nonlinear and complex, non-parametric machine-learning models were adopted for FSV estimation, as they can flexibly capture such patterns and provide robust predictive performance (Liu et al., 2022b; Wang et al., 2024c). Accordingly, this study selected six widely used machine-learning algorithms as base learners within the stacking ensemble framework to estimate the FSV of *Pinus kesiya* var. *langbianensis*.

Base Learner Algorithms

Random Forest (RF) (Breiman, 2001) is an ensemble machine-learning method that combines bootstrap aggregation (Bagging) with Classification and Regression Trees (CART). RF regression is well suited to predictive modeling, feature evaluation, and resistance to overfitting.

XGBoost is a tree-boosting machine-learning algorithm (Chen and Guestrin, 2016), that iteratively adds CART-based regression trees to improve predictive performance. It is computationally efficient, provides strong predictive performance, and can handle large-scale datasets effectively (Fang et al., 2022).

Gradient Boosting Decision Tree (GBDT) is an ensemble machine-learning algorithm that uses multiple decision trees as base learners (Zhang and Jung, 2019; Liang et al., 2020). Each new tree is constructed to place greater emphasis on samples that were poorly predicted by previous trees, so that the model is iteratively optimized for error correction (Friedman, 2001).

AdaBoost is an iterative ensemble algorithm that constructs a strong regressor by adjusting sample weights and combining multiple weak learners. It is well suited to relatively small datasets and is adaptable to different prediction tasks (Ba et al., 2024).

K-Nearest Neighbors (KNN) is a non-parametric lazy-learning algorithm that estimates a target value by identifying the K closest samples in feature space and averaging their observed outputs (Halder et al., 2024).

LightGBM is an efficient gradient-boosting framework for regression that is built upon the GBDT algorithm. It uses techniques such as histogram-based splitting to reduce training time and memory usage while maintaining strong predictive accuracy (Liang et al., 2020).

Stacking Algorithm

Stacking ensembles integrate predictions from multiple base learners to reduce individual model errors, thereby improving predictive accuracy and stability relative to single models (Fu et al., 2025; Jiang et al., 2025b). The architecture of the proposed stacking ensemble framework for FSV estimation is shown in **Figure 3**. A three-level stacking framework was implemented. At Level-0, the six base learners were trained using 10-fold cross-validation on the training data, and the resulting out-of-fold predictions were used to construct the meta-feature set. At Level-1, the predictions from the two best-performing base models were further integrated using meta-learners. At Level-2, a final meta-learner generated the final FSV predictions. To clarify the validation workflow, model development and internal comparison were based on cross-validation within the training data, whereas the final optimized stacking model was additionally evaluated on a hold-out test set. Because spatially blocked validation was not implemented in the current study, the reported accuracies may still be optimistic in the presence of spatial autocorrelation.

In this study, all machine-learning models were implemented using the Data Analysis-Machine Learning Regression module in SPSSPRO (https://www.spsspro.com). The corresponding hyperparameter settings are summarized in **Supplementary Table 1**.

### 2.4 Model assessment

Model assessment involved two complementary steps. During model development, 10-fold cross-validation was used within the training data for model comparison and internal performance assessment. After model selection, the optimized model was further evaluated on a hold-out test set that was not used during model fitting. Model performance was quantified using the coefficient of determination (R^2^; Equation (6)), root mean square error (RMSE; Equation (7)), and mean absolute error (MAE; Equation (8)) Because the current validation design was based on non-spatial resampling, these metrics should be interpreted as estimates of predictive performance under a non-spatial validation framework. Therefore, the reported accuracy metrics should be interpreted primarily as evidence for relative model comparison under the current validation design, rather than as definitive evidence of spatial transferability.

(6) $$\;R^{2}=1-\frac{\sum_{i=1}^{n}{\left(y_{i}-y_{i}\right)}^{2}}{\sum_{i=1}^{n}{\left(y_{i}-\bar{y_{i}}\right)}^{2}}$$

(7) $$RMSE=\sqrt{\frac{\sum_{i=1}^{n}{\left(y_{i}-\hat{y_{i}}\right)}^{2}}{n}}$$

(8) $$MAE=\frac{1}{n}\sum_{i=1}^{n}\left|y_{i}-\hat{y_{i}}\right|$$

In the formula, $y_{i}$ is the actual value of the target variable for the sample, $\hat{y_{i}}$ is the predicted value of the target variable from the model, and $\bar{y_{i}}$ is the mean value of the actual target variables for all samples.

## 3 Results

### 3.1 Semi-variogram analysis of LiDAR-derived metrics

The spot-level quality_flag variable, a binary indicator (1 or 0), was used exclusively for initial data quality screening and was not included among the predictors in the subsequent FSV estimation models. Following quality screening and feature selection, a final set of 14 predictor variables was retained for model development. The best-fitting theoretical variogram model and its corresponding parameters for each of these 14 variables are presented in **Table 4**.

**Table 4:** Optimal model and fitting parameters for GEDI characteristic variables.

Name	R²	RSS	Structural ratio (%)	Optimal model	Range (m)
Cover	0.555	6.27E-05	12.7%	Exponential model	2910
Fhd_normal	0.599	1.13E-03	14.3%	Exponential model	3600
Elevation	0.988	3.48	0.05%	Spherical model	12410
Pai	0.499	2.05E-03	12.8%	Exponential model	2850
Pgap_theta	0.554	6.30E-05	12.8%	Exponential model	2910
Rg	0.257	5061	7.4%	Exponential model	2700
Rh98	0.748	197	13.2%	Exponential model	3900
Rv	0.772	6514	13.5%	Exponential model	3840
Sensitivity	0.669	1.98E-09	13.2%	Exponential model	4500
Landsat_treecover	0.902	0.417	12.4%	Exponential model	3510
Leaf_off_doy	0.721	13387	12.7%	Exponential model	4680
Leaf_on_doy	0.782	17623	13.1%	Exponential model	5160
Modis_nonvegetated	0.633	54	12.4%	Exponential model	4860
Modis_treecover	0.904	0.273	15.0%	Exponential model	7200

**Table 4** shows that elevation is best fitted by a spherical variogram model, whereas the other variables are best fitted by exponential models. The fitted models yield R^2^ values ranging from 0.257 to 0.988, with most exceeding 0.50. However, all variables exhibited structural ratios lower than 25%. This indicates a very strong spatial correlation, suggesting that the distribution of variables in the system exhibits pronounced spatial dependence. Under such complex terrain conditions, OK may therefore be more susceptible to local bias and over-smoothed predictions.

### 3.2 Comparisons of spatial interpolation methods

To determine an appropriate number of SGCS realizations, simulations were conducted with N = 10, 25, 50, 75, 100, and 125 (Luo et al., 2023). Simulation convergence was monitored by tracking the coefficient of variation (CV) across all raster pixels for each variable. The simulation set at which the CV stabilized was selected to generate the final output realizations. Simulation was considered to have converged, and the corresponding N was regarded as sufficient, when the global CV stabilized between successive realization counts (Luo et al., 2024). The specific number of realizations used in the final output for each variable is summarized in **Table 5**.

**Table 5:** Comparisons of accuracy under different interpolation conditions.

Name	Number of simulations	R^2^					
		OK	SGCS	IDW	PSO-IDW	GS-IDW	GA-IDW
Cover	75	0.314	0.61	0.0962	0.0983	0.0962	0.0983
Fhd_normal	100	0.163	0.688	0.1515	0.1542	0.152	0.154
Elevation	125	0.932	0.919	0.911	0.920	0.919	0.920
Pai	50	0.284	0.481	0.241	0.241	0.241	0.241
Pgap_theta	100	0.106	0.113	0.124	0.125	0.124	0.125
Rg	100	0.137	0.621	0.135	0.140	0.142	0.142
Rh98	25	0.157	0.833	0.180	0.183	0.181	0.181
Rv	75	0.272	0.126	0.322	0.324	0.323	0.323
Sensitivity	75	0.176	0.389	0.169	0.171	0.170	0.170
Landsat_treecover	50	0.137	0.417	0.132	0.139	0.132	0.135
Leaf_off_doy	125	0.371	0.346	0.326	0.353	0.347	0.323
Leaf_on_doy	100	0.472	0.490	0.418	0.436	0.433	0.429
Modis_nonvegetated	50	0.342	0.523	0.318	0.330	0.329	0.329
Modis_treecover	75	0.534	0.320	0.507	0.517	0.514	0.508

**Figure 4:**
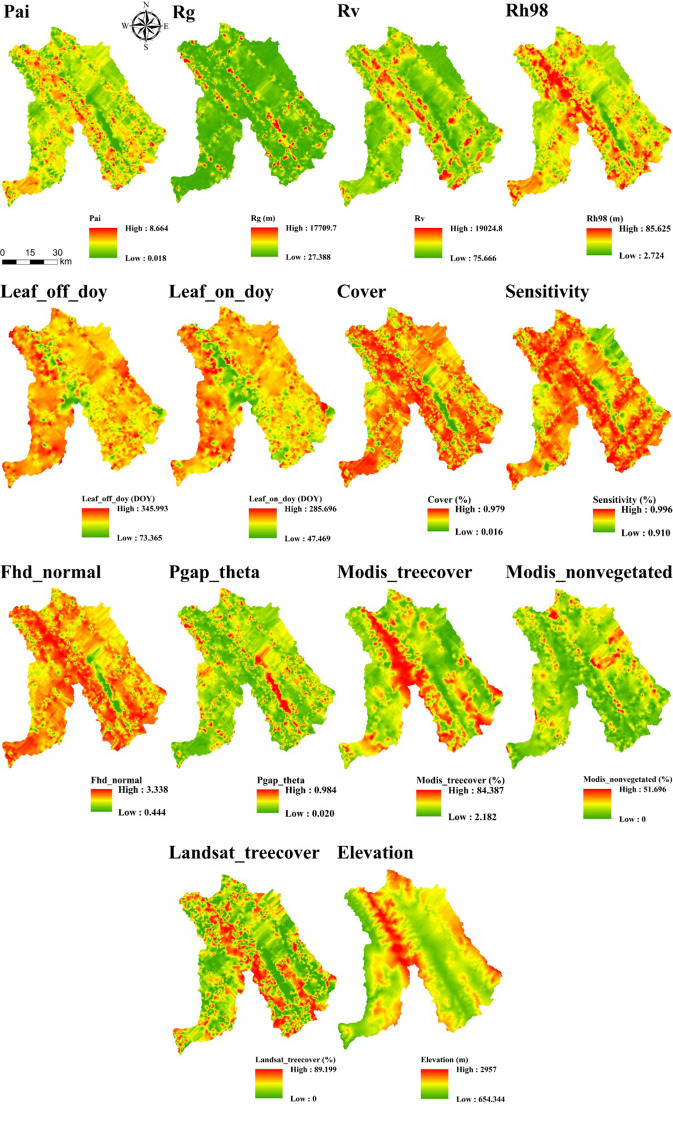
Results of OK interpolation of GEDI variables.

**Figure 5:**
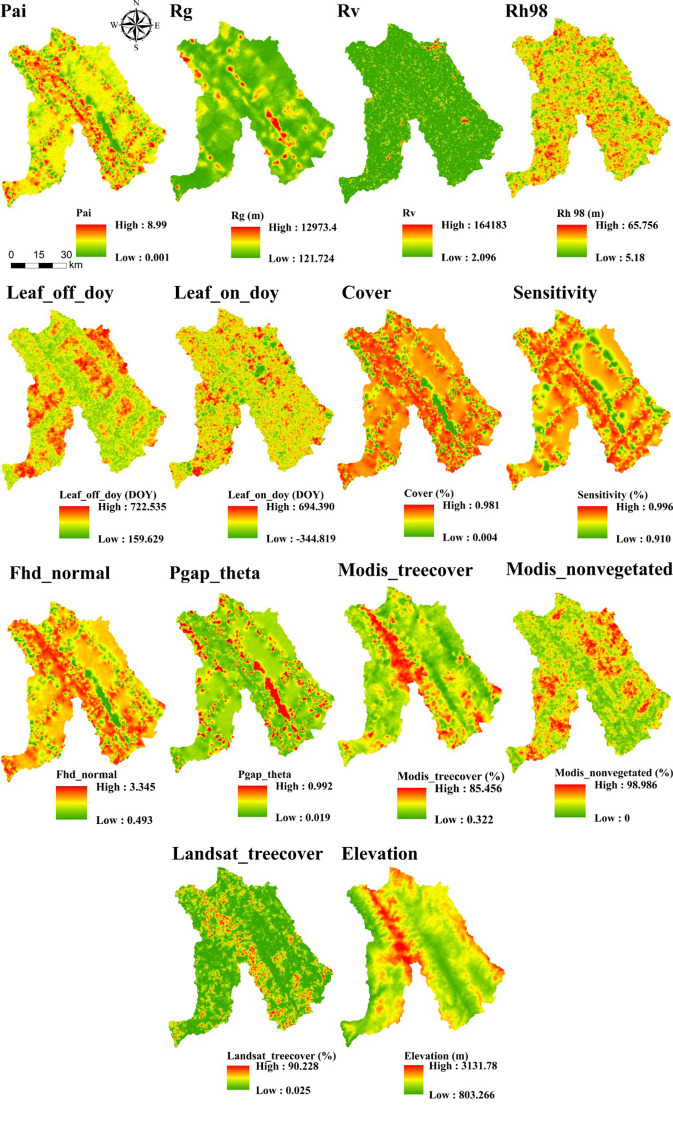
Results of SGCS interpolation of GEDI variables.

**Figure 6:**
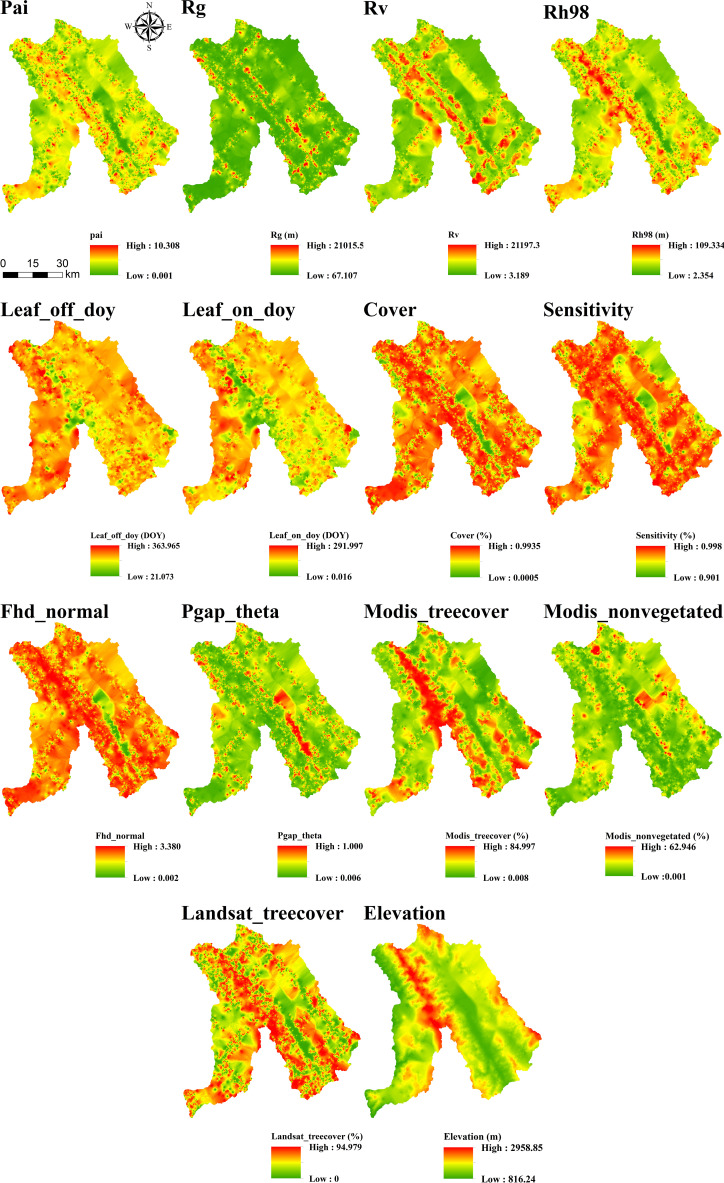
Results of IDW interpolation of GEDI variables.

**Figure 7:**
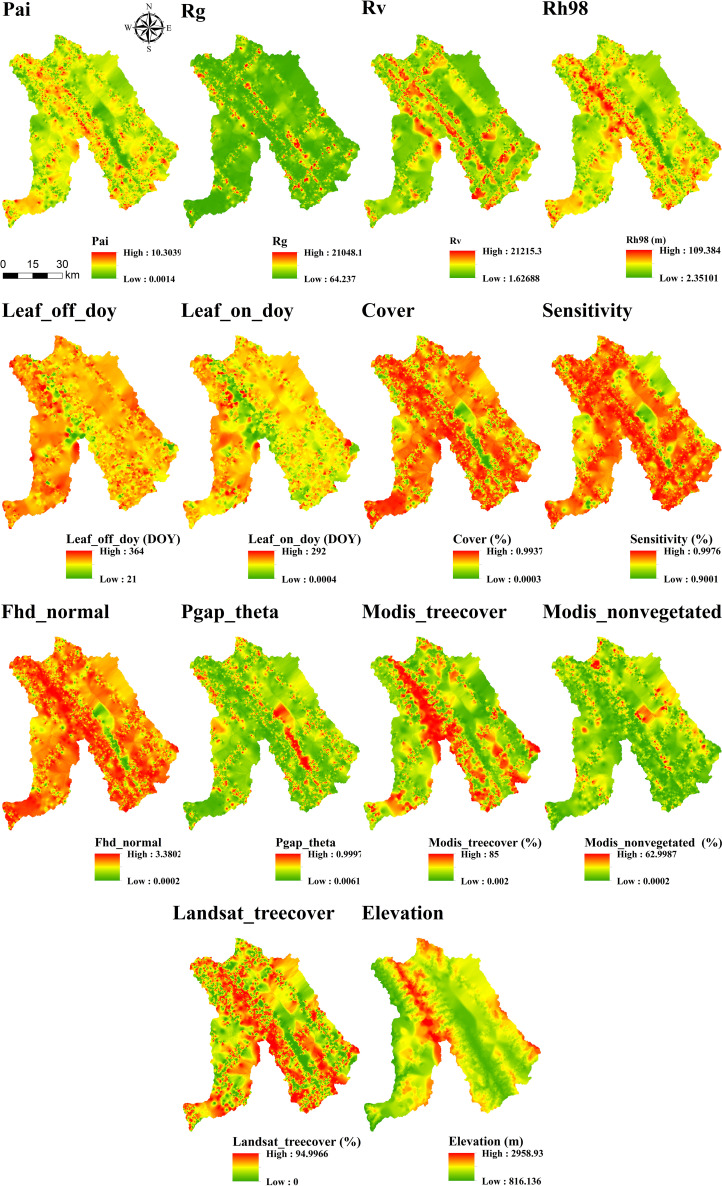
Results of PSO-IDW interpolation of GEDI variables.

**Figure 8:**
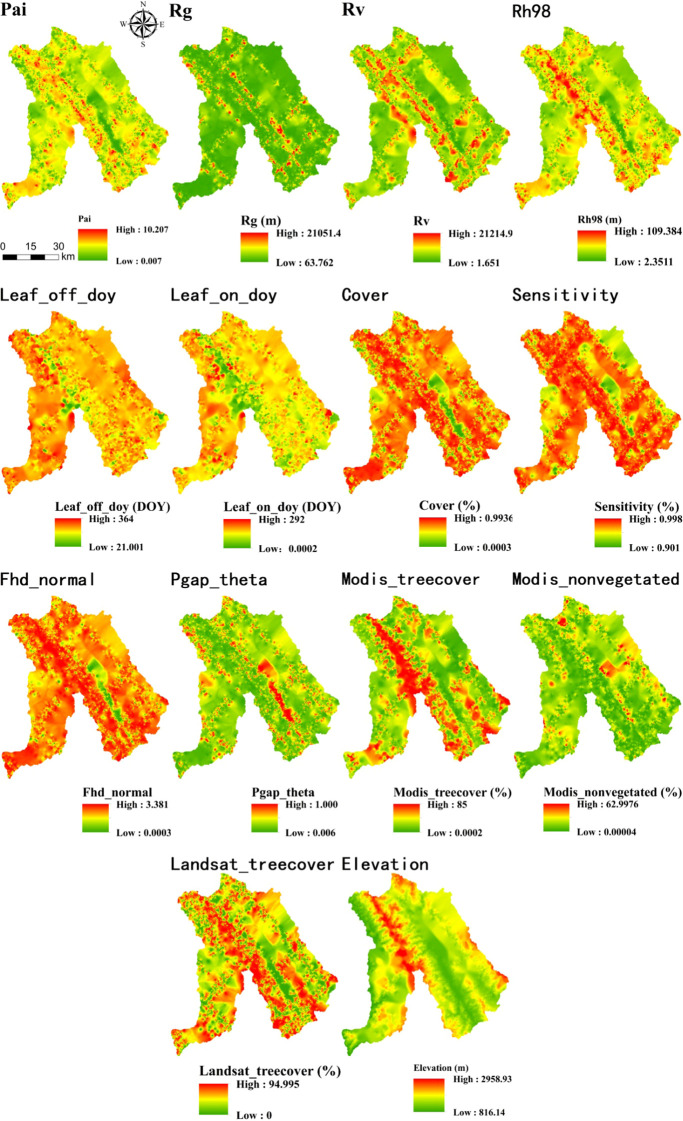
Results of GS-IDW interpolation of GEDI variables.

**Figure 9:**
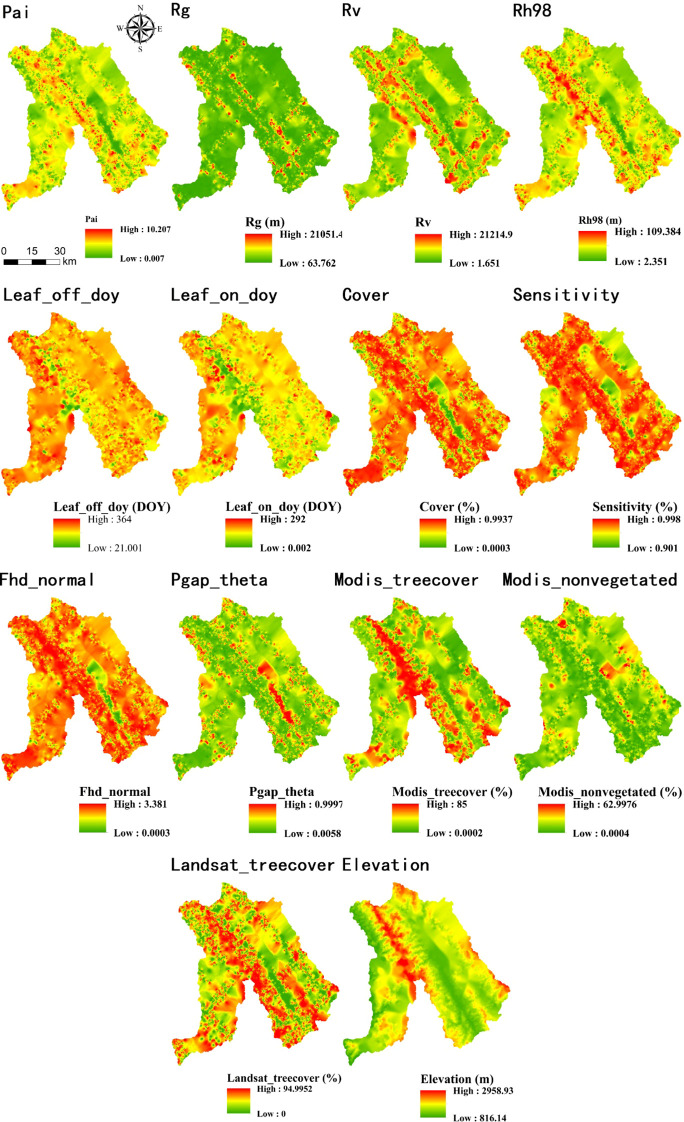
Results of GA-IDW interpolation of GEDI variables.

After comparing interpolation methods, SGCS achieved the highest R^2^ for most GEDI-derived predictors, particularly canopy-structure metrics (e.g., cover, Fhd_normal, Pai, Rg, and Rh98), indicating a stronger ability to reconstruct spatial variability from discrete footprints to continuous surfaces. In contrast, OK performed best for elevation, whereas several ancillary or phenology-related variables showed comparable performance across interpolation methods. IDW showed relatively good performance only for elevation, whereas its predictive accuracy was poor for the other variables. Based on the six interpolation methods, continuous raster surfaces were generated for the 14 GEDI-derived metrics. These interpolated products were then evaluated, and only variables with R^2^ ≥ 0.50 were retained for subsequent feature screening and MLSEM-based FSV prediction. Based on this interpolation assessment, SGCS was selected as the preferred footprint-to-surface approach because it showed the best performance for the key structural predictors retained for model construction. All interpolation results are presented in **Figures 4**–**9**.

### 3.3 Feature selection results

**Figure 10:**
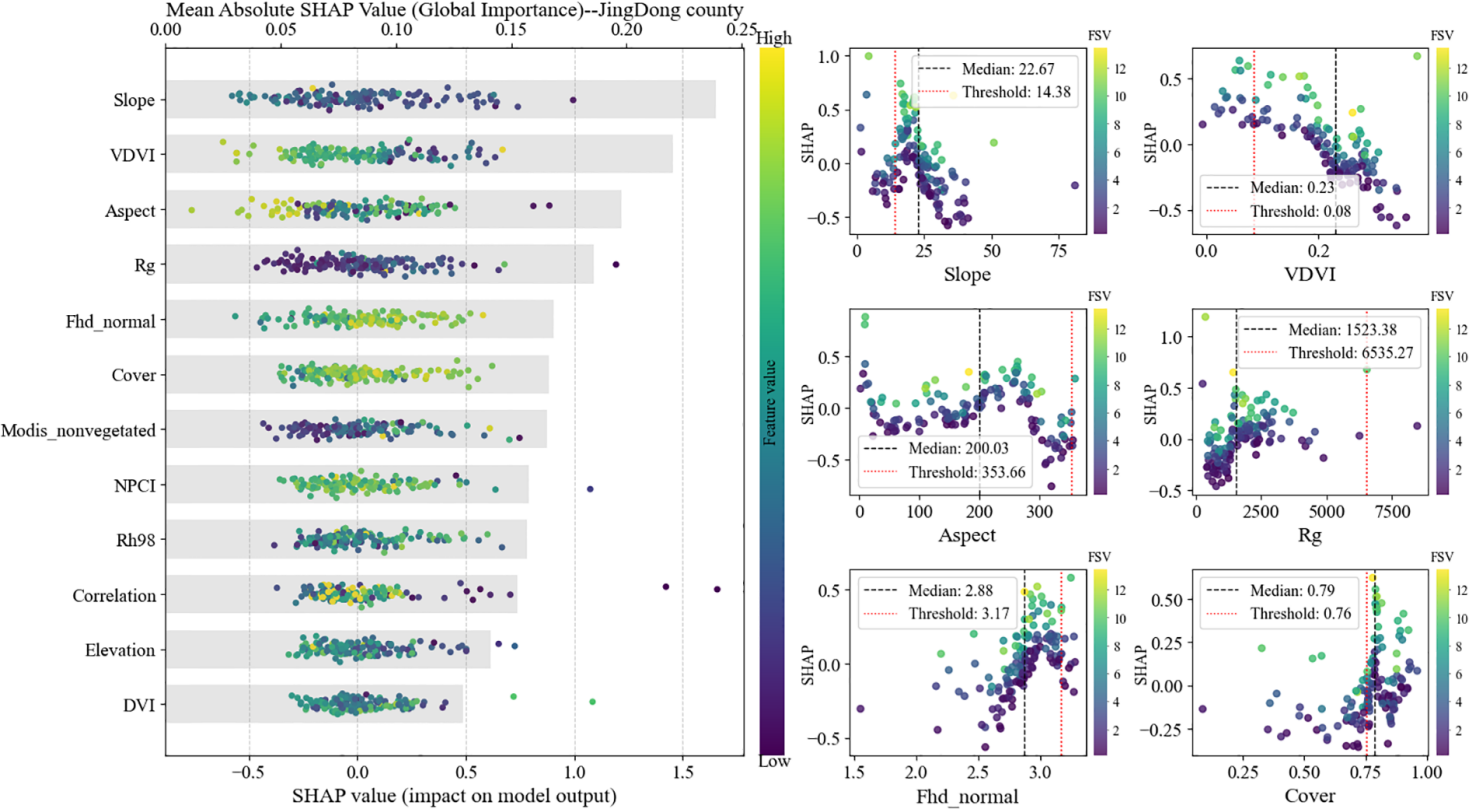
Results of SHAP analysis.

The relative importance of predictor variables for the FSV model, quantified using SHAP values, is shown in **Figure 10**. In total, 35 candidate predictors were assembled, including 6 GEDI metrics retained after SGCS-based spatial extrapolation, 3 topographic variables, 7 optical single-band reflectance variables, 11 vegetation indices, and 8 texture features. Multicollinearity was then reduced using an iterative VIF-based elimination procedure, in which predictors with VIF > 5 were removed stepwise and VIF values were recalculated after each removal until all remaining variables satisfied the criterion of VIF < 5 (details are provided in **Supplementary Table 2**). This process resulted in the retention of 12 predictors. Finally, SHAP-based ranking identified the six most influential predictors for FSV estimation: slope, aspect, VDVI, cover, Fhd_normal, and Rg.

### 3.4 Model performance and regional FSV mapping

**Figure 11:**
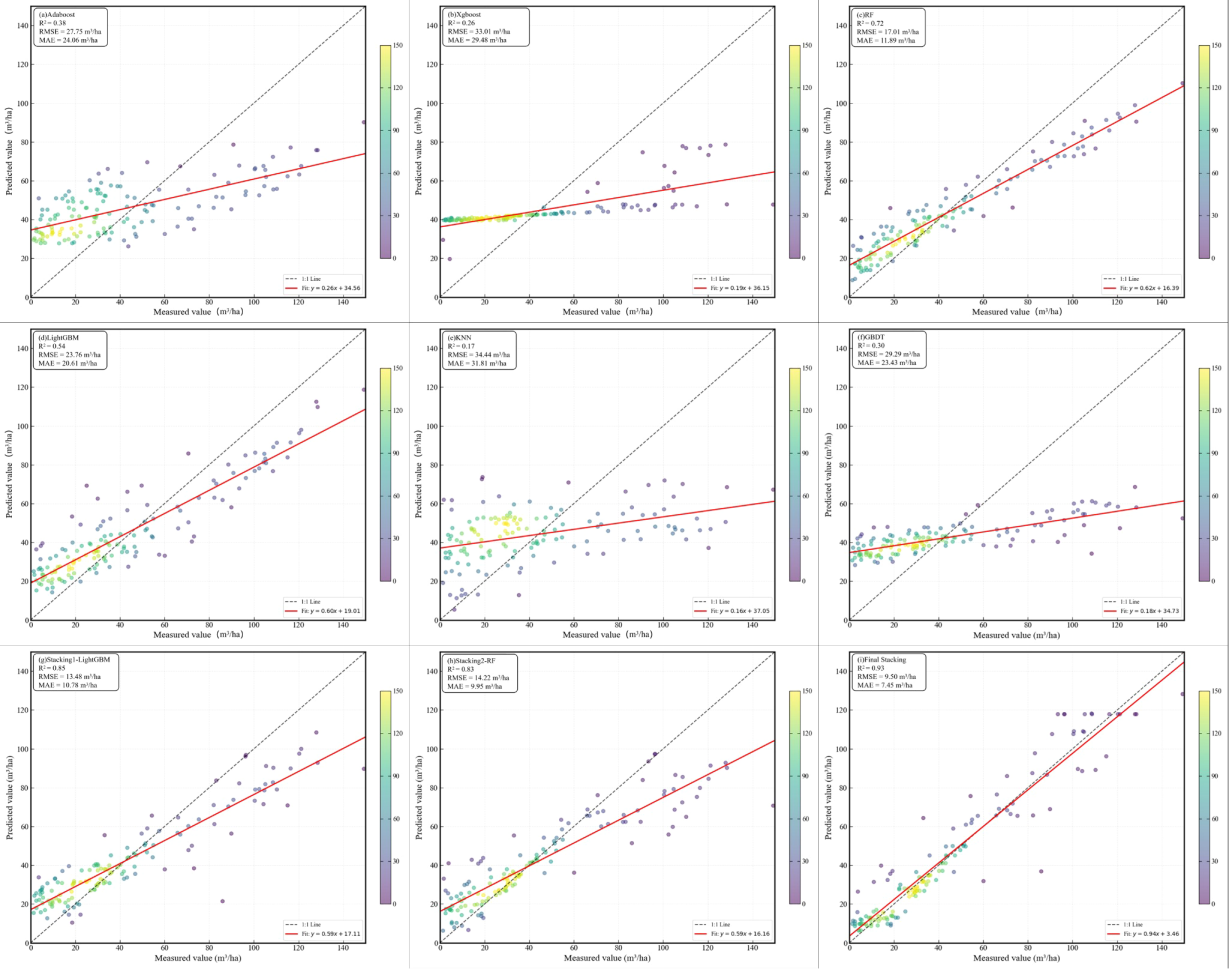
Scatterplots for different models. **(A)** is Adaboost; **(B)** is XGBoost; **(C)** is RF; **(D)** is LightGBM; **(E)** is KNN; **(F)** is GBDT; **(G)** is Stacking-1(LightGBM); **(H)** is Stacking-2(LightGBM); **(I)** is Final Stacking (LightGBM).

Among the base learners, RF achieved the highest predictive accuracy (R^2^ = 0.72, RMSE = 17.01 m^3^/ha) followed by LightGBM (R^2^ = 0.54, RMSE = 23.76 m^3^/ha). The first-level stacking ensembles, using RF and LightGBM as meta-learners, further improved predictive performance, with R² values increasing to 0.83-0.85. The final optimized MLSEM achieved the best overall performance (R^2^ = 0.93, RMSE = 9.50 m^3^/ha), outperforming all individual models and first-level stacking ensembles (**Figure 11**).

**Figure 12:**
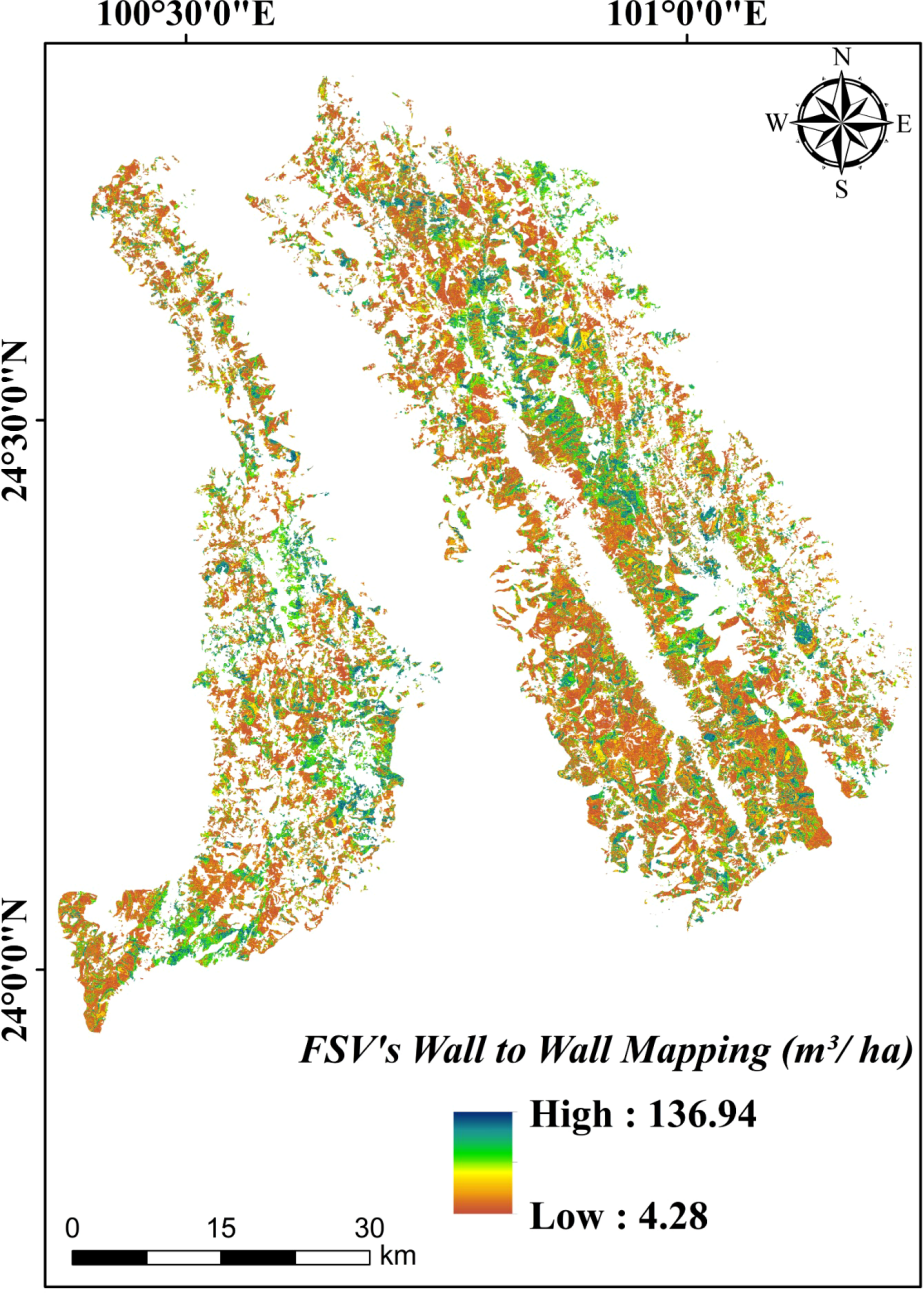
FSV of Pinus kesiya var. Langbianensis wall-to-wall mapping.

The optimized model was then applied to the SGCS-interpolated predictor surfaces to produce a wall-to-wall FSV map for 2019 (**Figure 12**). The estimated stand volume ranged from 4.28 to 136.94 m^3^/ha, with a mean of 43.89 m^3^/ha and a total standing stock of approximately 8.66 × 10^7^ m^3^ across the study area.

## 4 Discussion

### 4.1 FSV inversion performance and rationale for the indirect framework

This study adopted an indirect modeling framework in which GEDI metrics were first spatially extrapolated into continuous surfaces and then combined with optical and terrain predictors to estimate FSV using the stacked model. This framework differs from the more common direct, footprint-scale paradigm, in which field-measured FSV is regressed against co-located GEDI footprints (Demol et al., 2022; Xi et al., 2023). This choice reflects a practical trade-off between methodological rigor and regional applicability in rugged terrain. Direct footprint-scale modeling requires precise plot-footprint co-location, which is difficult to achieve in steep mountainous environments and can substantially reduce fieldwork efficiency and safety. More importantly, it would constrain the sampling design to the sparse and uneven distribution of GEDI footprints, thereby limiting the acquisition of regionally representative plots across topographic gradients and potentially introducing spatial sampling bias. By decoupling spatial extrapolation from model calibration, the indirect approach enables a more flexible and regionally representative and regionally representative field-sampling design that is not constrained by GEDI footprint geometry. Although this strategy introduces additional uncertainty during the footprint-to-surface transformation, it improves operational feasibility and spatial coverage for regional FSV assessment, particularly where GEDI observations are inherently sparse.

The mapped results suggest that this framework was effective for wall-to-wall FSV estimation in the study area. The mean mapped FSV was broadly consistent with the average of the field plots, indicating that the overall prediction level was reasonable. The estimated total standing stock differed substantially from the reported stock value (1.6×10^7^ m^3^). Therefore, this comparison should be interpreted cautiously and should not be treated as direct validation evidence. The discrepancy may reflect the combined effects of temporal harmonization, scale/support mismatch, interpolation uncertainty, and model extrapolation error. A key strength of the proposed framework is its practical and regional applicability. It supports wall-to-wall FSV mapping without restricting field sampling to GEDI footprints, thereby providing a useful spatial decision-support tool for inventory updating, management prioritization, and targeted field verification in complex mountainous forests. Moreover, GEDI-derived structural variables were not treated as fixed inputs; instead, interpolation effects were explicitly evaluated before hierarchical predictor integration, which enhances the methodological rigor of the framework and distinguishes it from studies based on a single interpolation method or simpler predictor-fusion strategies.

### 4.2 Spatial extrapolation of GEDI LiDAR metrics and uncertainty considerations

In this study, transforming sparse GEDI footprint metrics into wall-to-wall surfaces was a necessary step for regional FSV mapping because the indirect fusion framework required continuous structural predictors derived from discrete GEDI observations. Interpolation was therefore not only a technical preprocessing step, but also an important factor influencing the usefulness of GEDI-derived variables in subsequent modeling. Different interpolation methods impose different spatial assumptions, which can change the degree of smoothing and the preservation of local variability in the interpolated layers. These differences are especially important in mountainous environments, where strong spatial heterogeneity can amplify interpolation artifacts and distort ecologically meaningful structural variation.

Among the methods evaluated, deterministic approaches such as IDW and its optimized variants mainly rely on distance-decay smoothing. As a result, they may be less capable of representing the complex spatial structure of rugged terrain. By contrast, SGCS generates conditional realizations that honor the observed GEDI footprints while preserving spatial heterogeneity through stochastic simulation. In our results, SGCS better retained local structural variability and reduced the damping of local peaks, which likely improved the value of GEDI-derived predictors for subsequent FSV estimation. This interpretation is consistent with previous studies showing that conditional simulation can effectively characterize the spatial patterns of forest structural attributes (Jin et al., 2013; Luo et al., 2024). At the same time, interpolation performance should not be judged solely by reconstruction quality, because the ultimate goal is not only to reproduce footprint-level values, but also to generate predictor layers that support robust downstream FSV estimation.

Several additional sources of uncertainty may also have influenced the final mapping results. First, the stand volume used for model development was projected from 2016 to 2019 using a localized growth equation to reduce temporal mismatch between the field data and the remote-sensing predictors, rather than to represent directly measured 2019 field truth. Although the reported average relative error of the growth model was low (E = 0.064%), uncertainty introduced during this temporal harmonization process may still have propagated through the workflow. Specifically, because the projected 2019 FSV values were used as calibration targets, growth-model error would first affect the plot-level response variable, then influence model fitting and accuracy assessment, and may finally propagate into pixel-level FSV predictions and the regional total stock estimate. Second, the along-track sampling geometry of GEDI can produce striping artifacts and clustered footprint distributions. Although footprint thinning may reduce striping, it would also remove substantial local information and increase dependence on interpolation, potentially weakening local support in sparsely sampled areas. We therefore retained the full footprint set, while recognizing that residual striping and patch-like artifacts may still affect fine-scale spatial patterns. Third, we did not apply an additional empirical correction for horizontal displacement. Although GEDI L2B Version 2 has substantially improved geolocation performance and is considered suitable for many scientific applications that can tolerate moderate geolocation error (Tang et al., 2023), residual geolocation uncertainty may still influence interpolation results in spatially heterogeneous mountainous environments. Fourth, we did not apply an explicit topographic correction to the optical predictors, so terrain-induced illumination differences may still persist and may partly be confounded with vegetation signals. In addition, because texture is inherently scale-dependent (Ozkan and Demirel, 2021), the use of a single 5 × 5 GLCM window may not fully capture structural variation across spatial scales.

Taken together, these issues indicate that, although the proposed framework effectively constructed GEDI-based structural predictors under complex terrain conditions, further improvement is still needed to reduce uncertainty in mountainous forest applications. Future work should further assess uncertainty propagation from temporal harmonization, mitigate orbit-related artifacts through spatially balanced sampling or track-wise weighting, incorporate explicit topographic correction and multi-scale texture features, and quantify uncertainty by refitting and predicting across multiple SGCS realizations. More flexible spatial predictors based on machine learning or deep learning may also help capture nonstationary terrain effects, and track-related artifacts more effectively in future regional FSV mapping (Liu et al., 2022a; Wang et al., 2022).

### 4.3 Value of MLSEM and baseline interpretation

The proposed MLSEM combines multiple base learners through trained meta-models to exploit the nonlinear complementarities among GEDI-derived structural metrics, optical predictors, and terrain variables. In this architecture, the intermediate meta-learners transform potentially noisy outputs from the base learners into higher-level meta-features, while the final LightGBM meta-learner performs deeper nonlinear integration and bias correction. This hierarchical design is particularly relevant in the present study because several key predictors were derived from interpolated GEDI continuous surfaces and may therefore carry propagated interpolation uncertainty and local artifacts. Compared with the individual base learners and the first-level stacking models, MLSEM achieved superior predictive performance, highlighting the potential of hierarchical ensemble learning for FSV estimation in complex mountainous forests. This result is consistent with previous studies (Zhang et al., 2022; Liu et al., 2025), further indicating that stacking approaches can enhance the predictive stability of forest parameter estimation in complex environmental settings. In addition, Wang et al. (2025) demonstrated that a double-layer ensemble framework improved modeling performance in a complex geospatial classification task, further supporting the feasibility and effectiveness of hierarchical ensemble architectures in remote-sensing applications. Therefore, the contribution of the present study lies not only in applying a stacking framework, but also in explicitly linking GEDI interpolation strategy evaluation to downstream FSV prediction within a multi-level ensemble architecture for mountainous forests.

**Figure 13:**
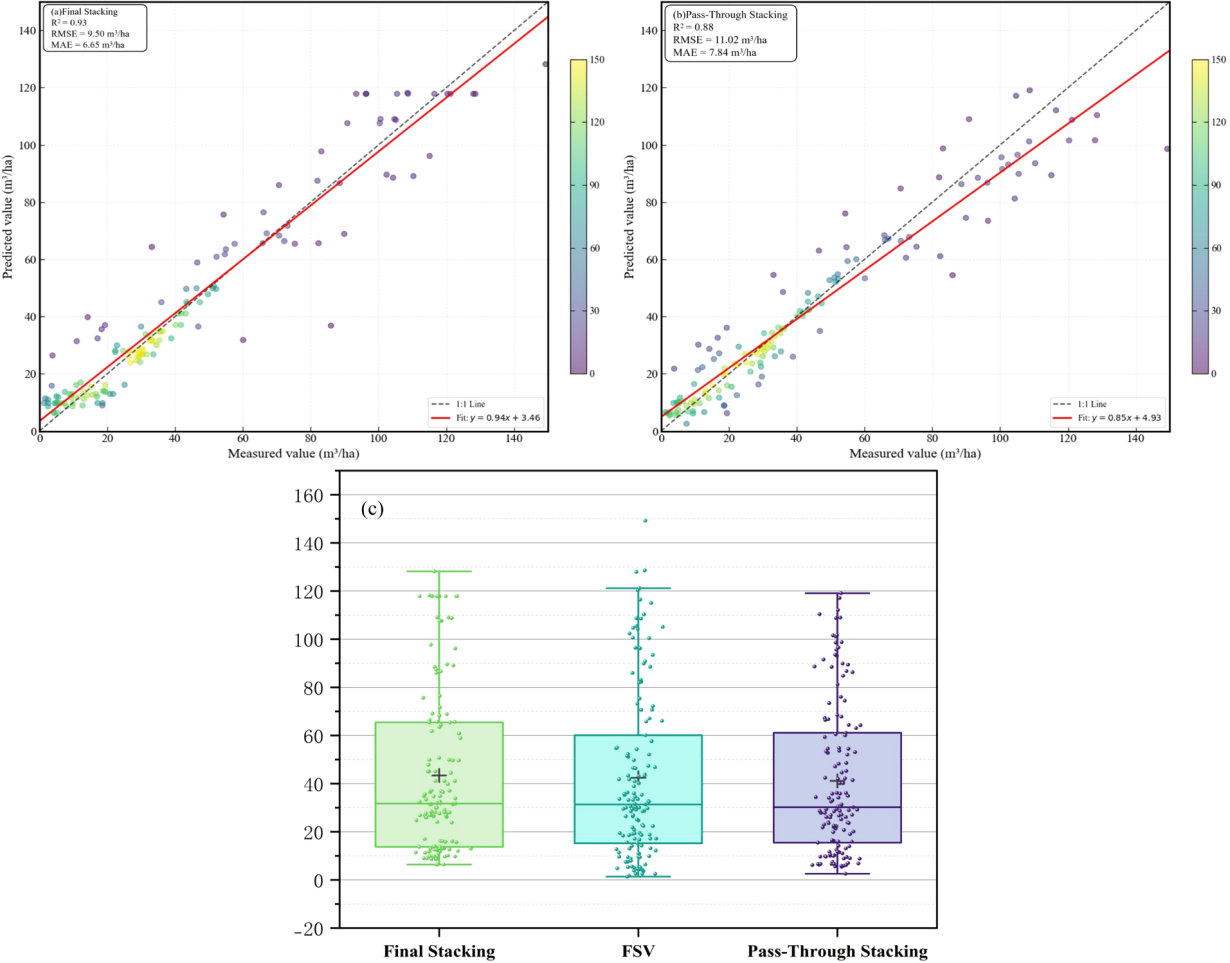
Comparison of the final stacking model and the pass-through baseline. **(A)** is Final Stacking (LightGBM); **(B)** is Pass-Through Stacking; **(C)** is Box plot comparison.

To further distinguish the contribution of the intermediate learning layer from that of architectural depth alone, we introduced a pass-through baseline model (**Figure 13**) as an additional control. Unlike MLSEM, the pass-through model performs only vector concatenation at the first level and does not apply any learnable nonlinear transformation. Its inferior performance relative to the final stacking model suggests that the advantage of MLSEM is more likely attributable to learnable nonlinear fusion and bias correction than to architectural depth alone. Moreover, MLSEM produced a wider and more ecologically plausible prediction range, allowing it to better capture high-FSV conditions, whereas the pass-through baseline tended to generate more strongly smoothed estimates that underrepresented spatial heterogeneity.

Although MLSEM outperformed the single models and the first-level stacking models, the lack of spatially blocked cross-validation means that optimistic bias arising from spatial autocorrelation may not have been fully eliminated. Future work should therefore adopt stricter nested out-of-fold prediction and spatially explicit blocking strategies to provide a more conservative and reliable evaluation of model generalization performance. More broadly, future studies could further refine hierarchical ensemble designs, test alternative meta-learning structures, and examine whether the integration of uncertainty-aware GEDI predictors can further improve the robustness of mountainous FSV estimation.

## 5 Conclusion

This study developed an integrated framework for regional FSV mapping of *Pinus kesiya* var. *langbianensis* by combining multi-source remote-sensing predictors, GEDI footprint-to-surface interpolation, and a multi-level stacking ensemble model. Three main conclusions can be drawn.

First, converting sparse GEDI footprints into continuous predictor layers is essential for indirect wall-to-wall FSV mapping in complex terrain. Among the tested interpolation methods, SGCS provided the most reliable continuous surfaces for several key canopy-structure variables and better preserved spatial heterogeneity than the deterministic alternatives.

Second, the proposed MLSEM improved predictive performance relative to the individual base learners and the simpler stacking baseline. In the current validation framework, RF was the best-performing single model (R^2^ = 0.72, RMSE = 17.03 m^3^/ha), whereas MLSEM achieved R^2^ = 0.93 and RMSE = 9.50 m^3^/ha. These results indicate the potential value of multi-level stacking for integrating GEDI structural metrics, optical variables, and topographic information.

Third, the optimized workflow generated a spatially explicit 2019 FSV map of *Pinus kesiya* var. *langbianensis* for Jingdong County, with an estimated total standing stock of approximately 8.66×10^7^ m^3^. Although this estimate differed from the reported total stock, the regional total should be interpreted cautiously because the discrepancy remains substantial. This difference highlights the need to consider temporal harmonization and model-related uncertainties when interpreting mapped stock totals.

Overall, the proposed framework combines practical regional applicability with methodological rigor, while still being subject to uncertainties associated with footprint-to-surface transformation and non-spatial validation; despite these limitations, it provides useful spatial decision support for inventory updating, management prioritization, and targeted field verification in complex mountainous forests.

## Data Availability

The data analyzed in this study is subject to the following licenses/restrictions: The field data used in this study are not publicly available because they involve information related to private landowners. These data are available from the corresponding author upon reasonable request. Requests to access these datasets should be directed to Qingtai Shu, shuqt@swfu.edu.cn.
