# Supplementary material for: Regional forest stock volume mapping using GEDI-based interpolation, multi-source remote sensing, and a multi-level stacking ensemble model in complex terrain
Source: Front Plant Sci. 2026 Apr 30;17:1827732. doi: 10.3389/fpls.2026.1827732 (PMC13171860; doi:10.3389/fpls.2026.1827732)
Supplement: Supplementary file 1 [file Table1.docx]

**Table S1.** Model key hyperparameter settings

| Model name | Parameter Settings |
| --- | --- |
| Adaboost | Loss: linear; n_estimators = 20; learning_rate = 0.05; max_depth = 5 |
| Xgboost | num_leaves = 20; max_depth = 4; min_child_samples = 1; min_split_gain = 2 |
| RF | n_estimators = 30; max_depth = 7; min_samples_split = 2, min_samples_leaf = 1 |
| LightGBM | Num_leaves = 20; max_depth = 10; min_child_samples = 1; min_split_gain=2; learning_rate=0.05 |
| KNN | Weights: uniform; metric: euclidean; algorithm:auto n_neighbors=5 |
| GBDT | n_estimators = 30; learning_rate = 0.05; subsample = 0.5; max_depth = 7; min_samples_leaf = 1 |

**Table S2.** Multicollinearity Test Results with Iterative Variable Elimination

| Removed Variable | VIF | Number of Remaining Variables |
| --- | --- | --- |
| B5 | 63742510967.27 | 34 |
| TVI | 17589634.21 | 33 |
| SAVI | 324868.80 | 32 |
| EVI | 110035.74 | 31 |
| NGBDI | 18768.49 | 30 |
| B4 | 7820.12 | 29 |
| B7 | 3071.88 | 28 |
| B2 | 1778.17 | 27 |
| Dissimilarity | 970.18 | 26 |
| RDVI | 594.22 | 25 |
| B1 | 337.32 | 24 |
| Entropy | 184.08 | 23 |
| B6 | 96.00 | 22 |
| Contrast | 34.49 | 21 |
| B3 | 14.35 | 20 |
| RVI | 9.51 | 19 |
| Homogeneity | 8.31 | 18 |
| Variance | 7.62 | 17 |
| Mean | 7.44 | 16 |
| ARVI | 6.83 | 15 |
| NDVI | 5.45 | 14 |
| Second Moment | 5.21 | 13 |
| DEM | 4.53 | 12 |
| VDVI | 3.52 | 11 |
| Cover | 2.99 | 10 |
| Fhd_normal | 2.80 | 9 |
| Rg | 2.01 | 8 |
| DVI | 1.95 | 7 |
| Correlation | 1.81 | 6 |
| Slope | 1.52 | 5 |
| NPCI | 1.46 | 4 |
| Aspect | 1.32 | 3 |
| Elevation | 1.31 | 2 |
| Modis_nonvegetated | 1.11 | 1 |
| Rh98 | 1.08 | Stop |
